# What's happening up north?

**DOI:** 10.1186/1757-1146-8-S2-P17

**Published:** 2015-09-22

**Authors:** Jason Warnock, Malindu Fernando

**Affiliations:** 1 Townsville Hospital and Health Service, Townsville, Qld, 4814, Australia

## Background

Research has investigated the motivators and deterrents which influence medical practitioners and nurses to consider rural placements within Australia; there is a scarcity in podiatry. The authors received funding from the North Queensland Regional Training Network (NQRTN) to investigate the views of podiatrists working in northern Australia, podiatry students and placement co-ordinators. This project, the Northern Australia Podiatry Clinical Placement Initiative (NAPCPI), was completed over 21 weeks during 2014. This project aimed to gather and analyse information on the current scope, need and factors of influence for increasing podiatry student placements to northern Australia.

## Methods

Two on-line Survey Monkey questionnaires were developed to identify the scope and capacity of student placements in northern Western Australia, Northern Territory and north Queensland. One survey was designed to evaluate information from podiatrists working in this region. Podiatrists were identified through professional networks and searches of publically available directories. A second survey was designed for podiatry students; this was distributed by university educators. Additional opinions were obtained through semi-structured interviews with university clinical coordinators. Results were published in a NQRTN report.

## Results

A total of 53 of the 96 identified podiatrists practising in northern Australia responded to the survey. Most participating podiatrists are based in large regional centres. 56% of respondents reported that they were working with other podiatrists. The podiatrists described their scope of practice and the models of service delivery. All except 5% of those surveyed were interested in providing supervision of students on placement; 81% were willing to undertake clinical placement training.

The student survey identified the motivators and deterrents for placements and the factors which would influence students to undertake placements in northern Australia. A total of 157 students replied, from seven podiatry programs. The cost of accommodation and travel were frequently reported as influencing factors, as well as having a podiatrist as the supervisor at the placement location.

The university interviews confirmed some of the student opinions however there was some variance to student thoughts relating to northern Australian placements.

## Conclusion

The project identified factors to incorporate into a model of podiatry clinical placements for northern Australia.

